# Optical coherence elastography measures the biomechanical properties of the *ex vivo* porcine cornea after LASIK

**DOI:** 10.1117/1.JBO.29.1.016002

**Published:** 2024-01-13

**Authors:** Achuth Nair, Fernando Zvietcovich, Manmohan Singh, Mitchell P. Weikert, Salavat R. Aglyamov, Kirill V. Larin

**Affiliations:** a University of Houston, Department of Biomedical Engineering, Houston, Texas, United States; b Pontificia Universidad Católica del Perú, Department of Engineering, Lima, Perú; c Baylor College of Medicine, Cullen Eye Institute, Houston, Texas, United States; d University of Houston, Department of Mechanical Engineering, Houston, Texas, United States; e Baylor College of Medicine, Department of Physiology and Biophysics, Houston, Texas, United States

**Keywords:** cornea, biomechanics, eye, optical coherence elastography, LASIK

## Abstract

**Significance:**

The biomechanical impact of refractive surgery has long been an area of investigation. Changes to the cornea structure cause alterations to its mechanical integrity, but few studies have examined its specific mechanical impact.

**Aim:**

To quantify how the biomechanical properties of the cornea are altered by laser assisted *in situ* keratomileusis (LASIK) using optical coherence elastography (OCE) in *ex vivo* porcine corneas.

**Approach:**

Three OCE techniques, wave-based air-coupled ultrasound (ACUS) OCE, heartbeat (Hb) OCE, and compression OCE were used to measure the mechanical properties of paired porcine corneas, where one eye of the pair was left untreated, and the fellow eye underwent LASIK. Changes in stiffness as a function of intraocular pressure (IOP) before and after LASIK were measured using each technique.

**Results:**

ACUS-OCE showed that corneal stiffness changed as a function of IOP for both the untreated and the treated groups. The elastic wave speed after LASIK was lower than before LASIK. Hb-OCE and compression OCE showed regional changes in corneal strain after LASIK, where the absolute strain difference between the cornea anterior and posterior increased after LASIK.

**Conclusions:**

The results of this study suggest that LASIK may soften the cornea and that these changes are largely localized to the region where the surgery was performed.

## 1 Introduction

The biomechanical properties of the cornea are an important biomarker for ocular health.^1^ Evaluating corneal rigidity can help diagnose corneal disease and monitor therapeutic efficacy because tissue biomechanical properties and structure are inextricably linked.^2^ This relationship has important implications for laser assisted *in situ* keratomileusis (LASIK), a surgical procedure for vision correction that involves structural modifications to the corneal stroma for refractive error correction.^3^ Modern LASIK involves the use of a femtosecond laser to create a lamellar corneal flap. Then, the flap is lifted, and an excimer laser is used to directly ablate the stromal bed. After ablation, the flap is repositioned on the surface of the cornea. Specific refractive errors can be corrected based on the selection of the ablation region. For example, treatment for myopia involves ablation of the center of the cornea, and treatment for hyperopia involves ablation of the corneal mid-periphery. Such significant changes to the corneal structure have consequences for corneal biomechanical properties.^4^ In rare cases, patients who have undergone LASIK may experience ectasia as the procedure can compromise corneal biomechanical and structural integrity.^5^^,^^6^ To minimize the risk of ectasia, the inclusion criteria for this refractive procedure are conservatively based on the structural properties of the cornea, including thickness and topography. However, these geometric parameters do not account for corneal stiffness, which plays an important role during early ectasia development.^7^^,^^8^ The next generation of customized refractive surgery may incorporate biomechanical mapping in addition to structural imaging, for a more personalized surgical treatment.^9^

Despite the necessity of corneal biomechanical assessments, there are very few clinical tools that can measure these properties. Air-puff tonometry-based techniques, such as the Corvis ST and the ocular response analyzer, have been repeatedly used to measure corneal biomechanical properties after LASIK. However, these techniques can only measure corneal stiffness by inducing large, non-physiological displacements,^10^^,^^11^ which limits their ability to map corneal biomechanical properties and measure quantitative biomechanical parameters, e.g., Young’s modulus. Optical elastography techniques, including Brillouin microscopy and optical coherence elastography (OCE), have both been used to measure the biomechanical properties of the cornea after refractive surgery^12^^,^^13^ with significantly fewer limitations. While Brillouin microscopy has shown distinct changes in corneal stiffness due to refractive surgery, this technique cannot natively provide sample structural information,^14^ and understanding the relationship between the Brillouin modulus and Young’s modulus is still an area of investigation.^14^^,^^15^

In this work, we examined the changes in mechanical properties in the cornea after LASIK in *ex vivo* porcine eyes. Three OCE modalities were used, including wave-based air-coupled ultrasound OCE (ACUS-OCE),^16^ compression OCE,^17^ and heartbeat OCE (Hb-OCE).^18^ ACUS-OCE provides an estimate of shear modulus across the corneal tissue, and both Hb-OCE and compression OCE provide a similar measurement in the axial direction. Combined, these techniques provide insight into the complex changes in the mechanical properties of the cornea before and after refractive surgery.

## 2 Methods

**Fig. 1:**
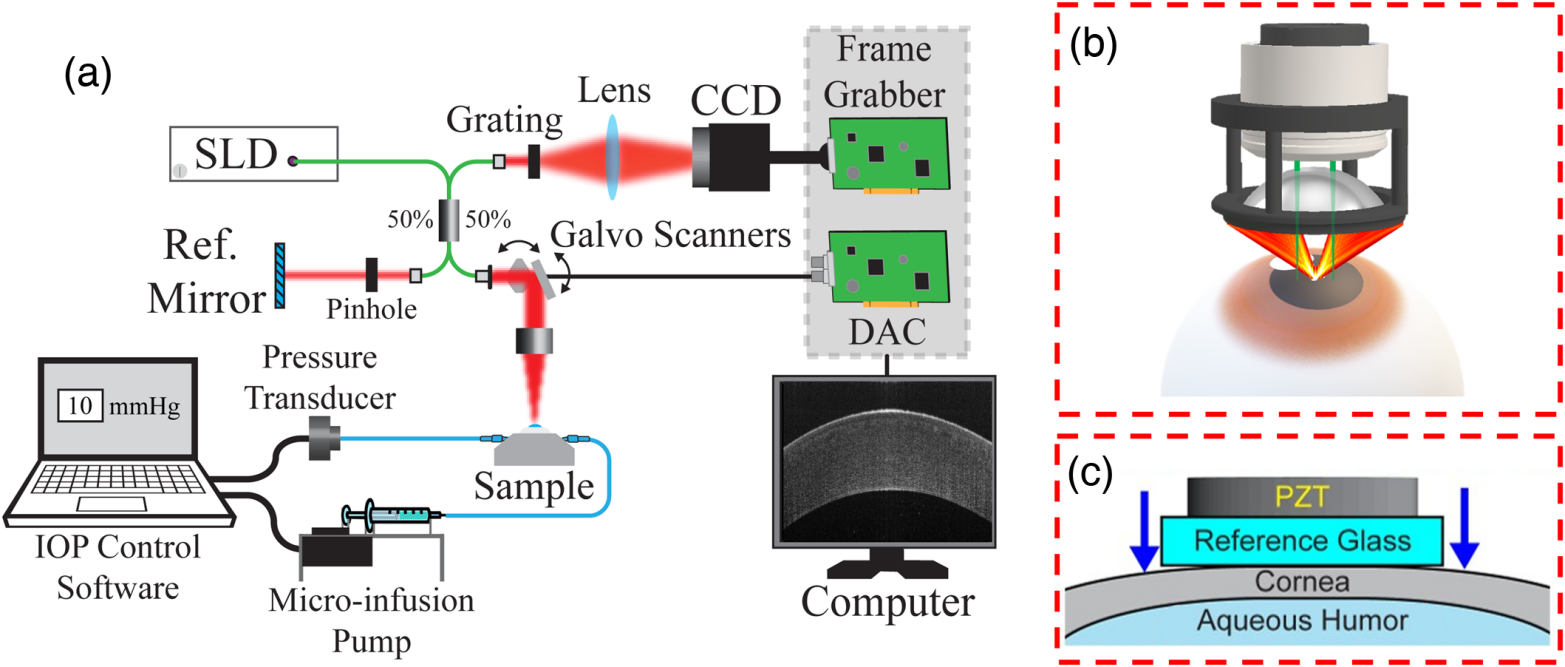
(a) OCE system schematic. Hb-OCE is performed without any additional components. (b) ACUS-OCE sample arm configuration with ACUS transducer co-focused with objective lens. (c) Compression OCE sample arm in common path configuration. Note that for compression OCE, the reference arm shown in (a) is blocked.

Six pairs of whole porcine eye globes were shipped within 24 h of enucleation (Sioux-Preme Packing Co., Sioux Center, Iowa). One eye of each pair was kept as a control, and LASIK was performed on the fellow eye. The treated eyes were mounted in a custom-built eye holder, and intraocular pressure (IOP) was maintained using a closed-loop IOP controller.^19^ A LASIK flap was created with a diameter of 8.5 mm and thickness of 110  μm (Intralase iFS150, Johnson & Johnson Vision, Irvine, California). The flap was pulled back, and the stromal bed was ablated with the excimer laser (VISX Star S4, Johnson & Johnson Vision, Irvine, California) with a maximum diameter of 6.5 mm. The ablation depth was set to 130  μm to simulate a refractive treatment of approximately –10 diopters. Following the completion of the excimer ablation, the LASIK flap was replaced in the usual fashion. OCE imaging was performed immediately after LASIK on all eye globe pairs. Each eye globe was mounted in a custom-built eye holder, and OCE measurements were taken as IOP was controlled using the closed-loop IOP controller. Measurements were performed using an SD-OCT system with a 25 kHz A-line rate, 9  μm axial resolution, and 8  μm lateral resolution. The system had a displacement stability of <1  nm in the common path configuration and ∼20  nm in the dual-arm configuration. Three types of OCE techniques were performed, including ACUS-OCE, Hb-OCE, and compression OCE. Three pairs were measured using ACUS-OCE, and the remainder were measured using both the Hb-OCE and compression OCE techniques. Due to time constraints and tissue degradation, not all eyes could be measured by each method. Figure 1 shows a summary schematic for each technique. Sample hydration was maintained with 1× phosphate-buffered saline.

### 2.1 ACUS-OCE

ACUS-OCE was performed as described in our previous works.^20^^,^^21^ Briefly, a spherically focused ACUS transducer with a central resonance frequency of 1 MHz, ∼34  mm diameter, and ∼10  mm diameter opening was co-focused with the OCT sample beam and mounted ∼20  mm away from the surface of the cornea. Wave excitation was performed using five cycles of a 2 kHz square pulse (50% duty cycle) amplitude modulating a 1 MHz sinusoidal signal. This signal was then amplified using a 53 dB power amplifier (A150; Electronics & Innovation, Rochester, New York) before driving the ACUS transducer.^20^^,^^21^

OCE measurements were taken in M-B-mode configuration at 25 kHz A-line scan rate with M-scan length of 1000 A-lines acquired over time at each imaging position. The ACUS-induced displacement produced at the corneal apex resulted in the propagation of a Lamb wave across the cornea. The wave propagation was sampled over 500 positions along an 8 mm line across the center of the cornea. Each measurement took ∼20  s to complete. The mechanical wave speed was estimated by tracking the peak of the axial particle velocity propagating across the scan region. Measurements were acquired at 10, 20, and 30 mmHg for both the untreated and LASIK-treated eyes.

### 2.2 Hb-OCE

Hb-OCE was performed on a separate set of three pairs of eyes. Imaging was performed in B-M-mode across the central 6 mm of the cornea with a B-scan width of 1000 A-lines and a frame rate of ∼20  Hz. Data acquisition was performed as the eye globe underwent a sinusoidal fluctuation of 1 mmHg peak to peak amplitude and 10 s period at baseline IOP of 10 and 20 mmHg, respectively. Each acquisition took 30 s for up to three IOP cycles. Fluctuations in IOP cause corneal compression and expansion, which can be captured by measuring the phase difference between consecutive OCT images. OCT phase images were denoised using the vector method,^22^ and the resulting denoised phase data were converted to real displacement after two-dimensional unwrapping.^23^ Displacement data were translated to strain using the weighted least squares method.^24^ The cumulative sum of inter-frame strains was calculated to determine the total strain change relative to the initial image with zero displacement. A detailed description of the Hb-OCE acquisition and processing methods can be found in our previous works.^18^^,^^25^

### 2.3 Compression OCE

The same set of eyes that was used for Hb-OCE was also used for compression OCE. A reference glass mounted to a piezoelectric ring actuator (HPSt 150/14-10/12 Piezomechanik GmbH, Munich, Germany) was aligned with the OCT beam in the common path configuration. A small amount of mineral oil was added to the surface of the cornea for lubrication, and the reference glass was gently lowered down onto the cornea until a 6 mm region of the tissue was applanated. OCT image acquisition followed the same method as in Hb-OCE. Images were acquired in B-M-mode across the center of the cornea at ∼20  Hz frame rate with a B-scan width of 1000 A-lines. The actuator was synchronized to the OCT frame trigger, such that one image was acquired while the cornea was compressed, and the consecutive image was acquired in the uncompressed state. Measurements were performed at 10 and 20 mmHg IOP. Data processing followed a similar methodology as the Hb-OCE technique; motion was detected between consecutive frames (compressed and uncompressed) using the OCT phase difference, the phase difference images were denoised using the vector method, the denoised data were converted to the displacements, and the displacements were translated to strain. Unlike the Hb-OCE method, total tissue displacement occurred between consecutive B-scans, so that inter-frame strain was equivalent to peak-to-peak strain in Hb-OCE. A thorough description of the compression OCE data acquisition and processing workflow can be found in our previous research.^17^^,^^26^

## 3 Results

**Fig. 2:**
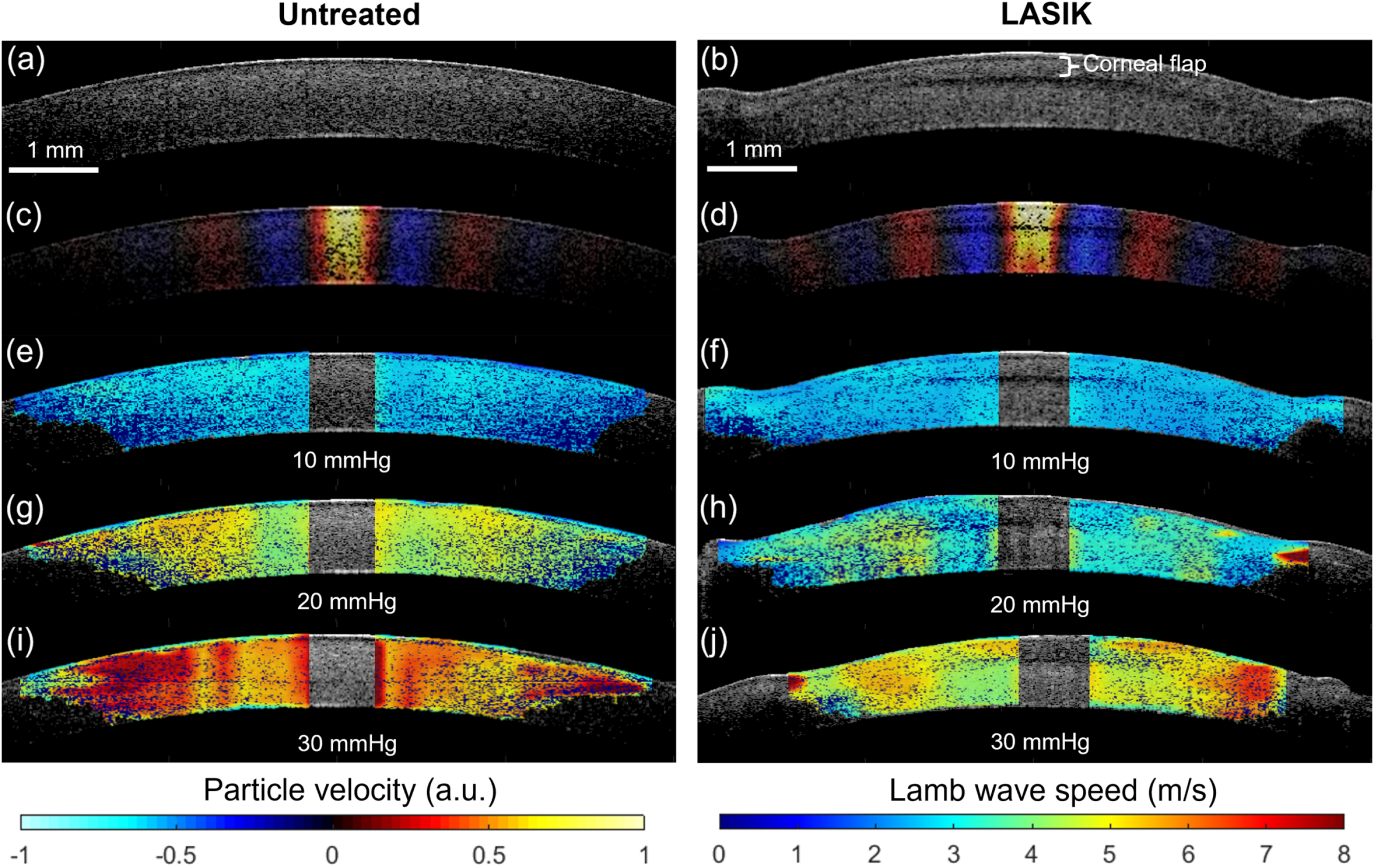
(a), (b) OCT structural B-mode of untreated and LASIK treated porcine cornea, respectively. (c), (d) Wave propagation map for each tissue type. (e), (g), (i)  Wave speed elastogram for untreated porcine cornea at 10, 20, and 30 mmHg, respectively. (f), (h), (j) Wave speed elastogram for LASIK treated porcine cornea at 10, 20, and 30 mmHg, respectively.

Figures 2(a) and 2(b) illustrate representative OCT images of an untreated and LASIK-treated cornea, respectively. The corneal flap and the demarcation line showing the incision region are clearly visible. Laser ablation resulted in a visible lateral inhomogeneity and a thickness loss in the central corneal region. Figures 2(c) and 2(d) show the wave propagation map (axial particle velocity) for the untreated and LASIK-treated corneas, respectively, at a given moment in time after the excitation. A guided Lamb wave is clearly seen propagating across the cornea. The increased magnitude of particle velocity in the LASIK-treated cornea suggests a decreased stiffness in the tissue, as the ACUS excitation energy was the same between measurements. Figures 2(e), 2(g), 2(i) and 2(f), 2(h), 2(j) show the wave speed map at 10, 20, and 30 mmHg for the untreated and LASIK-treated corneas, respectively. The elastogram shows a marked increase in wave speed as a function of IOP, consistent with previous work.^21^^,^^27^^,^^28^ However, the wave speed is notably lower in the LASIK-treated corneas compared to the untreated tissue at the same IOP.

**Fig. 3:**
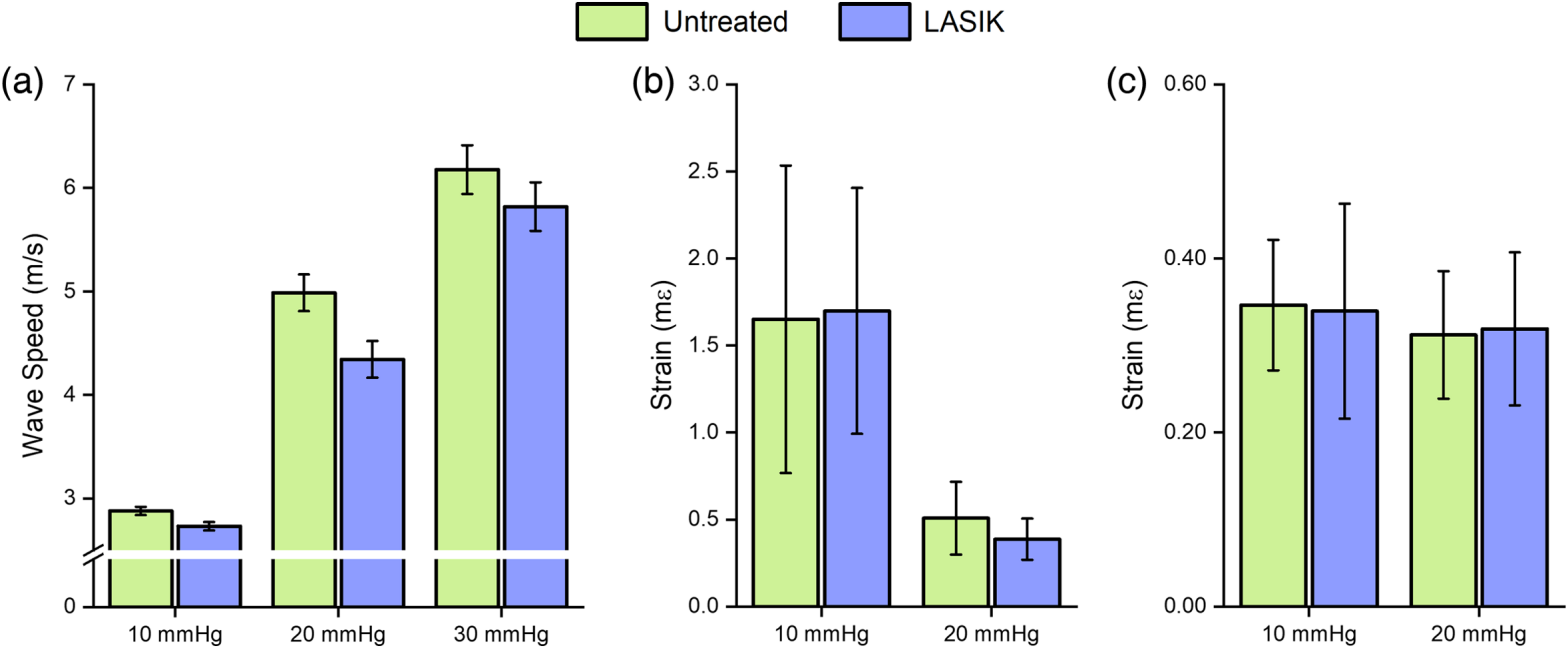
(a) Lamb wave speed as a function of IOP, measured by ACUS-OCE. (b) Strain change as a function of IOP measured using Hb-OCE for untreated and LASIK corneas. (c) Strain change as a function of IOP measured using compression OCE for untreated and LASIK corneas.

Mean wave speeds for the untreated corneas were 2.88±0.04, 4.99±0.18, and 6.18±0.24  m/s at 10, 20, and 30 mmHg IOP, respectively. Mean wave speeds for the LASIK-treated corneas were 2.73± 0.05, 4.34±0.57, and 5.82±0.97  m/s at 10, 20, and 30 mmHg, respectively. Analysis of all samples showed a statistically significant increase in wave speed as a function of IOP for both the untreated (df=1, F=22.6, p<0.001) and the LASIK treated (df=1, F=16.6, p<0.001) corneas by one way ANOVA. Corneas treated with LASIK had a lower wave speed compared to the untreated samples, suggesting a decrease in stiffness due to the treatment, but this relationship was marginally not statistically significant (p=0.06 by Wilcoxon signed ranks test) and may also be influenced to loss of corneal thickness during LASIK. Figure 3(a) summarizes the ACUS-OCE results.

**Fig. 4:**
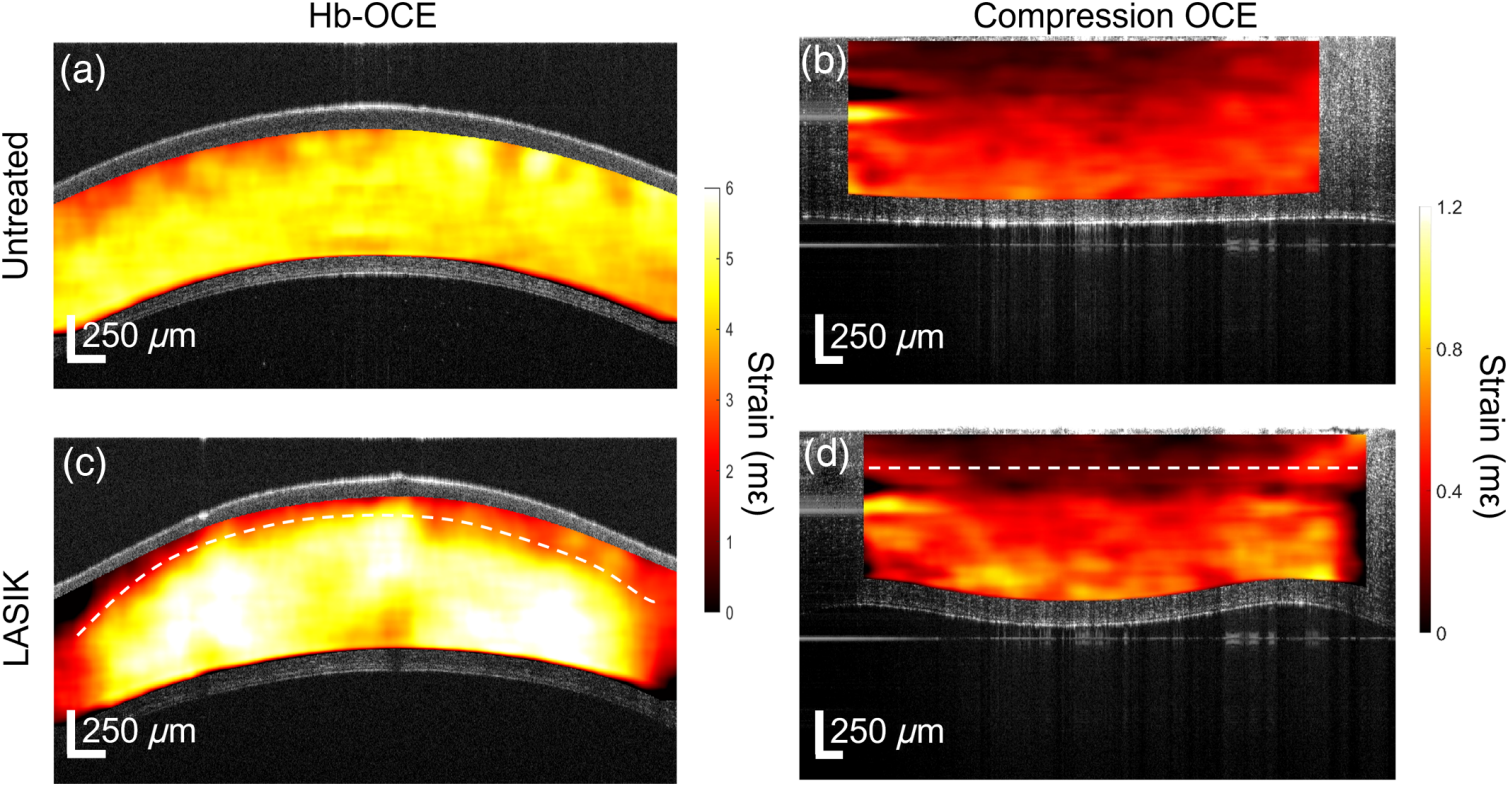
(a), (b) Hb-OCE and compression OCE strain maps for untreated porcine corneas. (c), (d) Hb-OCE and compression OCE strain maps for LASIK treated porcine corneas. The dashed white line represents the incision region for the LASIK treated corneas.

**Fig. 5:**
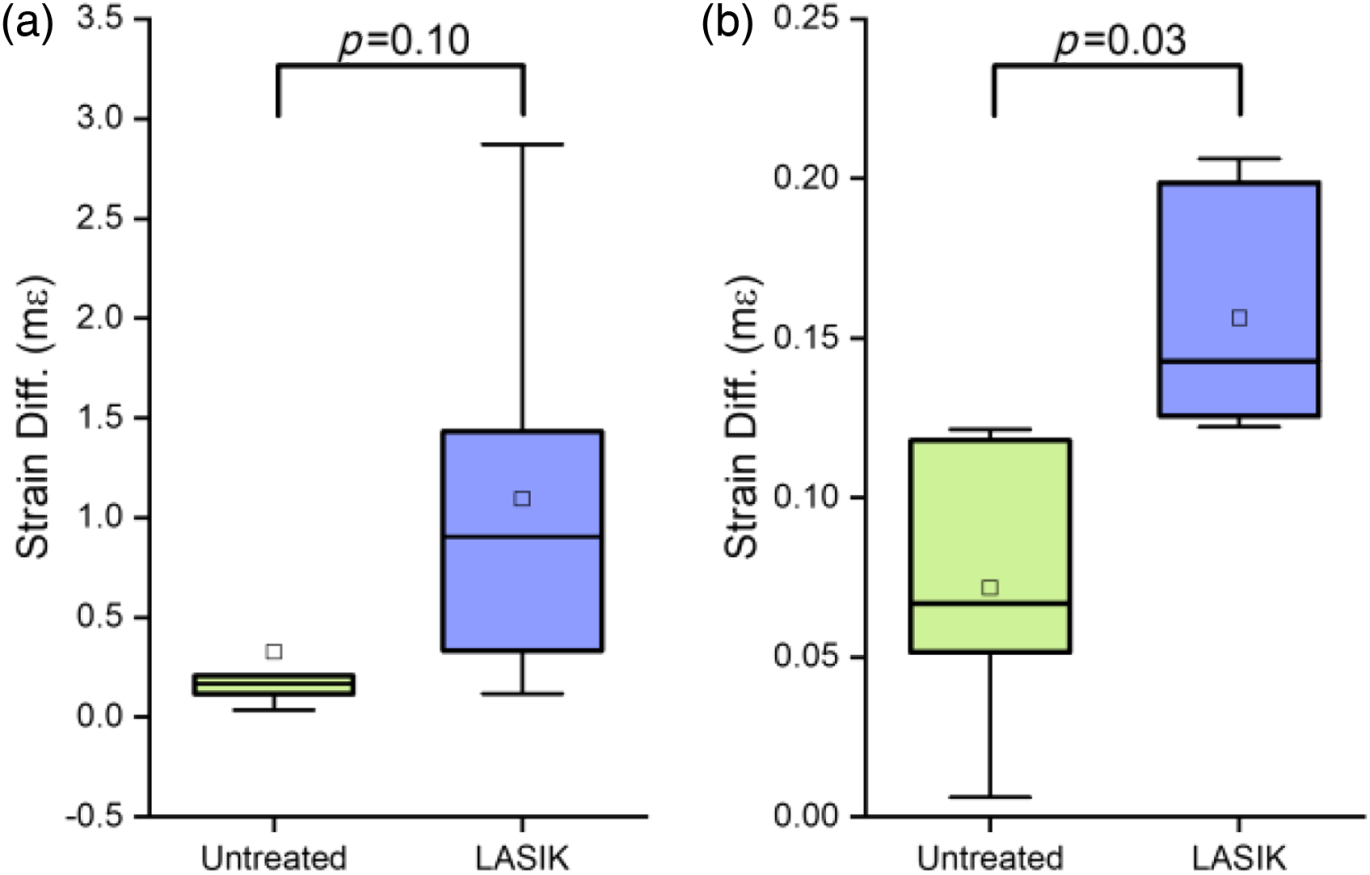
(a) Absolute strain difference between the anterior and posterior cornea as measured by Hb-OCE. P-value of 0.10 measured by Wilcoxon signed ranks test. (b) Absolute strain difference between the anterior and posterior cornea as measured by compression OCE. P-value of 0.03 calculated by Wilcoxon signed ranks test.

Hb-OCE and compression OCE were performed on the same samples (n=3) since the data could be acquired with the same imaging setup. Measurements were performed only at 10 and 20 mmHg for both techniques due to experimental time constraints and sample degradation. Representative samples for untreated and LASIK are shown in Fig. 4. Note that both techniques measure axial strain (i.e., along the cornea optical axis; millistrain: mε) and an increase in strain is a decrease in stiffness. However, Hb-OCE induced larger amplitude displacements as compared to compression OCE, leading to larger strains. Figures 3(b) and 3(c) illustrate that both Hb-OCE and compression OCE did not show a statistically significant difference in strain between 10 and 20 mmHg (p=0.37 for untreated measured by Hb-OCE, p=0.18 for untreated measured by compression OCE, p=0.18 for LASIK measured by Hb-OCE, and p=0.42 for LASIK measured by compression OCE using Wilcoxon signed-rank test), likely due to small sample size and mild sample degradation. However, both methods showed a distinct difference in localized strain from the anterior stroma compared to the posterior. For both techniques, the cornea was segmented through the midline between the epithelium and endothelium. The absolute value of the difference between anterior and posterior strain was quantified for each sample. A box-and-whisker plot of the result is shown in Fig. 5. Mean strain difference measured by Hb-OCE for the untreated and LASIK-treated corneas was 0.33±0.44  mε and 1.09±0.99  mε, respectively. The mean strain difference measured by compression OCE for the untreated and LASIK-treated corneas was 0.07±0.04  mε and 0.16±0.04  mε, respectively. While both techniques appeared to show a difference in stiffness, only compression OCE showed a statistically significant difference in regional strain after LASIK surgery (p=0.1 for Hb-OCE, p=0.03 for compression OCE, Wilcoxon signed ranks test), likely due to limitations in sample size, delays between surgery and measurement, and changes in tissue stiffness and geometry due to hydration.

## 4 Discussion

In this work, three OCE techniques were used to measure the stiffness of paired porcine corneas, where one eye was left untreated and the other underwent LASIK surgery. The Lamb wave speed measurement provided by ACUS-OCE can provide a reasonable estimate of the shear modulus of the tissue.^29^ The compressive strain measured by Hb-OCE and compression OCE can both provide information regarding axial Young’s modulus.^30^ Both the quasistatic and the dynamic techniques measure distinct aspects of corneal elasticity, and all three techniques independently suggest that the LASIK procedure may cause a reduction in corneal stiffness. LASIK involves laser incision and ablation of the corneal stroma, directly compromising the component of the cornea with the greatest biomechanical contribution, i.e., the stroma. While some research suggests that the Bowman’s membrane contributes to corneal tensile strength,^4^ the OCE methods used here lack the resolution to distinguish the mechanical contribution of this thin layer. The measured reduction in stiffness supports what is expected based on the mechanism of LASIK. For example, the reduction in Lamb wave speed correlated with LASIK-induced damage to the collagen lamellae within the tissue, which compromised its shear modulus. That damage to the lamellae may have also caused a disruption to the axial Young’s modulus of the cornea, as suggested by the change in strain measured by Hb-OCE and compression OCE. In the untreated tissue, the anterior and posterior regions of the stroma responded uniformly to axial compression. However, after LASIK, the corneas showed a distinct increase in strain difference, suggesting that the procedure causes the corneal flap and residual stromal bed to respond separately to axial compression. While this behavior may be because the flap is not bound to the stromal bed in this *ex vivo* study, it does suggest that Hb-OCE and compression OCE may be able to measure regional differences in mechanical properties caused by refractive procedures. Furthermore, regional assessment of mechanical properties, as seen in this study, may provide additional insight regarding the specific mechanism of complications such as LASIK-induced ectasia.

While these preliminary results are promising, there are some major limitations to this work. Primarily, this study was performed *ex vivo* and illustrated only the immediate biomechanical impact of the surgery on the cornea. However, the study sheds no light on the mechanical alterations in the tissue during and after the healing process. Nevertheless, OCE may be able to evaluate changes in mechanical properties throughout the wound healing process^31^ and is a direction of our future work. In this study, the corneal flap was laid flat against the residual stromal bed, and no external method for adherence was utilized. In a typical LASIK procedure, the flap adheres to the residual stromal bed naturally and begins to seal within a short period of time in part due to the pumping function of the corneal endothelium.^32^ As the tissue begins to heal, its integrity may recover, but an overall softening is still expected.^4^

Another major limitation of this study is the limited sample size. While the results of this study may suggest that there is a difference between the two groups, the limited sample size that was used in this study prevents any thorough conclusions. Furthermore, in this study, contralateral symmetry between fellow eyes was assumed; the untreated fellow eye was thought to be biomechanically similar to the treated eye. However, future studies will directly examine corneal stiffness changes after LASIK by studying the same eye before and after treatment. Despite these limitations, here we lay the groundwork for future evaluations of corneal stiffness changes after refractive surgery procedures.

In addition, there are several tested models for translating Lamb wave speed to viscoelastic properties, which have been implemented in OCE for the cornea. The complex boundary conditions of the LASIK-treated corneas that were present in this work make a thorough analysis of viscoelasticity more complex.^33^^,^^34^ Han et al. described a Lamb wave model for estimating the Young’s modulus and viscosity of the cornea. However, this model does not account for the multilayer structure of the tissue, particularly in the case of the LASIK-treated samples, where the corneal stroma has been sliced and ablated; meaning that the tissue geometry does not meet the basic requirements of the model. Future work will focus on establishing a robust, multilayer model that more accurately represents LASIK-treated corneas. However, it should be noted that the exaggerated layered structure that we see in our results is limited to the *ex vivo* case. In the case of clinical LASIK, since the flap and residual stromal bed rapidly bond together, a multilayer model applicable for the *ex vivo* case may not reflect the corneal geometry and boundary conditions in clinical LASIK.

In our previous work, we have shown that Hb-OCE and compression OCE had nonsignificant differences in strain *in vivo*.^26^ Both techniques provide similar information despite the distinct differences between each technique. However, here, we did not note a similarity in strain measured by the two techniques, likely due to the distinct differences in displacement amplitude between the physical compression by the mechanical actuator in compression OCE and the 1 mmHg IOP peak-to-peak amplitude displacement in Hb-OCE. Hb-OCE measurements were performed with a dramatically higher strain compared to compression OCE, enabling us to estimate corneal stiffness at different regions along the stress–strain curve. Based on trends from our previous work, we expect that the differences between the techniques measured here would be much smaller had measurements been performed *in vivo*, since the corneal displacement measured *in vivo* was dramatically lower than the displacement induced here in the Hb-OCE measurements. In our previous work, we quantified the stiffness of the cornea using Hb-OCE and compression OCE by assessing the ratio of IOP change to corneal strain. However, the difference in the magnitude of IOP change between both methods (1 mm peak to peak for Hb-OCE and 0.2 peak to peak for compression OCE^17^) ensures that the stiffness measured will not be comparable between both methods, and the lack of an appropriate model that accounts for ocular geometry and stress distribution in the tissue makes comparison to Young’s modulus estimation obtained from wave-based OCE difficult as well. Future work will focus on the development of appropriate models to estimate Young’s modulus given these parameters.

The results of this work suggest that LASIK has an impact on the biomechanical properties of the cornea, potentially compromising cornea structural and mechanical integrity. Other refractive procedures likely cause similar changes to the cornea. However, procedures such as photorefractive keratectomy or small incision lenticule extraction, which have no or significantly smaller incisions in the corneal stroma, may maintain corneal biomechanical integrity without compromising refractive surgery outcomes.^13^ Our future work will further examine the biomechanical impact of various refractive surgery procedures using optical elastography procedures.

## 5 Conclusion

In this work, three OCE techniques, ACUS-OCE, Hb-OCE, and compression OCE, were used to assess the mechanical properties of paired corneas, where one sample was left untreated and the other underwent LASIK. All three techniques showed that LASIK caused a reduction in corneal stiffness, though in the limited number of samples. In addition, Hb-OCE and compression OCE demonstrated the changes in elasticity distribution with depth after LASIK. Future work will examine changes in corneal biomechanical properties *in vivo* after LASIK and other refractive procedures.

## Data Availability

The data presented in this work was available upon reasonable request.

## References

[1] Kling S. Hafezi F., “Corneal biomechanics: a review,” Ophthal. Physiol. Opt. 37(3), 240–252 (2017). OPOPD5 0275-5408 10.1111/opo.12345 28125860

[2] Roberts C. J. Dupps W. J. Downs J. C., Biomechanics of the Eye, Kugler Publications (2018).

[3] Reinstein D. Z. Archer T. J. Gobbe M., “The history of LASIK,” J. Refractive Surg. 28(4), 291–298 (2012). 10.3928/1081597X-20120229-01 22496438

[4] Dupps W. J. Jr. Wilson S. E., “Biomechanics and wound healing in the cornea,” Exp. Eye Res. 83(4), 709–720 (2006). EXERA6 0014-4835 10.1016/j.exer.2006.03.015 16720023 PMC2691611

[5] Comaish I. F. Lawless M. A., “Progressive post-LASIK keratectasia: biomechanical instability or chronic disease process?” J. Cataract Refractive Surg. 28(12), 2206–2213 (2002). 10.1016/S0886-3350(02)01698-X 12498861

[6] Binder P. S. et al., “Keratoconus and corneal ectasia after LASIK,” J. Refractive Surg. 21(6), 749–752 (2005). 10.3928/1081-597X-20051101-15 16329368

[7] Ambrósio R. Jr. et al., “Evaluation of corneal shape and biomechanics before LASIK,” Int. Ophthalmol. Clin. 51(2), 11–38 (2011). IOPCAV 0020-8167 10.1097/IIO.0b013e31820f1d2d 21383577

[8] Zvietcovich F. et al., “Multi-meridian wave-based corneal optical coherence elastography in normal and keratoconic patients,” ARVO Annual Meeting, 2380–A0183 (2022). IOVSDA 0146-0404

[9] Roberts C., “Biomechanics of the cornea and wavefront-guided laser refractive surgery,” J. Refractive Surg. 18(5), S589–592 (2002). 10.3928/1081-597X-20020901-18 12361163

[10] Pepose J. S. et al., “Changes in corneal biomechanics and intraocular pressure following LASIK using static, dynamic, and noncontact tonometry,” Am. J. Ophthalmol. 143(1), 39–47.e31 (2007). AJOPAA 0002-9394 10.1016/j.ajo.2006.09.036 17188041

[11] Yang E. Roberts C. J. Mehta J. S., “A review of corneal biomechanics after LASIK and SMILE and the current methods of corneal biomechanical analysis,” J. Clin. Exp. Ophthalmol. 6(6), 507 (2015). 10.4172/2155-9570.1000507

[12] Randleman J. B. Su J. P. Scarcelli G., “Biomechanical changes after LASIK flap creation combined with rapid cross-linking measured with Brillouin microscopy,” J. Refractive Surg. 33(6), 408–414 (2017). 10.3928/1081597X-20170421-01 28586502

[13] Zhao Y. et al., “Quantitative evaluation of *in vivo* corneal biomechanical properties after SMILE and flex surgery by acoustic radiation force optical coherence elastography,” Sensors 23(1), 181 (2022). SNSRES 0746-9462 10.3390/s23010181 36616779 PMC9823345

[14] Ambekar Y. S. et al., “Multimodal imaging system combining optical coherence tomography and Brillouin microscopy for neural tube imaging,” Opt. Lett. 47(6), 1347–1350 (2022). OPLEDP 0146-9592 10.1364/OL.453996 35290310 PMC9088521

[15] Prevedel R. et al., “Brillouin microscopy: an emerging tool for mechanobiology,” Nat. Methods 16(10), 969–977 (2019).1548-7091 10.1038/s41592-019-0543-3 31548707

[16] Zvietcovich F. et al., “Confocal air-coupled ultrasonic optical coherence elastography probe for quantitative biomechanics,” Opt. Lett. 45(23), 6567–6570 (2020). OPLEDP 0146-9592 10.1364/OL.410593 33258863 PMC10041740

[17] Singh M. et al., “Compressional optical coherence elastography of the cornea,” Photonics 8 (4), 111 (2021). 10.3390/photonics8040111 37727230 PMC10508915

[18] Nair A. et al., “Heartbeat OCE: corneal biomechanical response to simulated heartbeat pulsation measured by optical coherence elastography,” J. Biomed. Opt. 25(5), 055001 (2020). JBOPFO 1083-3668 10.1117/1.JBO.25.5.055001 32372574 PMC7199791

[19] Twa M. D. et al., “Spatial characterization of corneal biomechanical properties with optical coherence elastography after UV cross-linking,” Biomed. Opt. Express 5(5), 1419–1427 (2014). BOEICL 2156-7085 10.1364/BOE.5.001419 24877005 PMC4026912

[20] Zvietcovich F. et al., “*In vivo* assessment of corneal biomechanics under localized cross-linking treatment using confocal air-coupled optical coherence elastography,” Biomed. Opt. Express 13(5), 2644–2654 (2022). BOEICL 2156-7085 10.1364/BOE.456186 35774330 PMC9203097

[21] Nair A. et al., “Multiple optical elastography techniques reveal the regulation of corneal stiffness by collagen XII,” Investig. Ophthalmol. Visual Sci. 63(12), 24–24 (2022). IOVSDA 0146-0404 10.1167/iovs.63.12.24 PMC9680591 36383352

[22] Matveyev A. et al., “Vector method for strain estimation in phase-sensitive optical coherence elastography,” Laser Phys. Lett. 15(6), 065603 (2018).1612-2011 10.1088/1612-202X/aab5e9

[23] Ghiglia D. C. Romero L. A., “Robust two-dimensional weighted and unweighted phase unwrapping that uses fast transforms and iterative methods,” J. Opt. Soc. Am. A 11(1), 107–117 (1994). JOAOD6 0740-3232 10.1364/JOSAA.11.000107

[24] Kennedy B. F. et al., “Strain estimation in phase-sensitive optical coherence elastography,” Biomed. Opt. Express 3(8), 1865–1879 (2012). BOEICL 2156-7085 10.1364/BOE.3.001865 22876350 PMC3409705

[25] Nair A. et al., “Heartbeat optical coherence elastography: corneal biomechanics *in vivo*,” J. Biomed. Opt. 26(2), 020502 (2021). JBOPFO 1083-3668 10.1117/1.JBO.26.2.020502 33624461 PMC7901857

[26] Nair A. et al., “Multimodal heartbeat and compression optical coherence elastography for mapping corneal biomechanics,” Front. Med. 9, 833597 (2022). FMBEEQ 10.3389/fmed.2022.833597 PMC9037093 35479957

[27] Kirby M. et al., “Optical coherence elastography in ophthalmology,” J. Biomed. Opt. 22(12), 121720 (2017). JBOPFO 1083-3668 10.1117/1.JBO.22.12.121720 29275544 PMC5745712

[28] Singh M. et al., “Ultra-fast dynamic line-field optical coherence elastography,” Opt. Lett. 46(19), 4742–4744 (2021). OPLEDP 0146-9592 10.1364/OL.435278 34598188 PMC9121022

[29] Zvietcovich F. Larin K., “Wave-based optical coherence elastography: the 10-year perspective,” Progr. Biomed. Eng. 4(1), 012007 (2021). PRBEEZ 0920-5438 10.1088/2516-1091/ac4512 PMC8856668 35187403

[30] Zaitsev V. Y. et al., “Strain and elasticity imaging in compression optical coherence elastography: the two-decade perspective and recent advances,” J. Biophotonics 14(2), e202000257 (2021). 10.1002/jbio.202000257 32749033

[31] Mekonnen T. et al., “Longitudinal assessment of the effect of alkali burns on corneal biomechanical properties using optical coherence elastography,” J. Biophotonics 15(8), e202200022 (2022). 10.1002/jbio.202200022 35460537 PMC11057918

[32] Bissen-Miyajima H. et al., “Role of the endothelial pump in flap adhesion after laser in situ keratomileusis,” J. Cataract Refractive Surg. 30(9), 1989–1992 (2004). 10.1016/j.jcrs.2004.01.045 15342067

[33] Han Z. et al., “Optical coherence elastography assessment of corneal viscoelasticity with a modified Rayleigh-Lamb wave model,” J. Mech. Behav. Biomed. Mater. 66, 87–94 (2017). 10.1016/j.jmbbm.2016.11.004 27838594 PMC5182155

[34] Pitre J. J. et al., “Nearly-incompressible transverse isotropy (NITI) of cornea elasticity: model and experiments with acoustic micro-tapping OCE,” Sci. Rep. 10(1), 12983 (2020). SRCEC3 2045-2322 10.1038/s41598-020-69909-9 32737363 PMC7395720

